# Homemade plasmid Miniprep solutions for affordable research in low-fund laboratories

**DOI:** 10.1186/s13568-022-01483-x

**Published:** 2022-11-01

**Authors:** Mohamed A. Elnagar, Mohamed F. Ibrahim, Magdy Albert, Maya M.Talal, Mahmoud M. Abdelfattah, Ehab El-Dabaa, Reham Helwa

**Affiliations:** 1 Biotechnology program, Faculty of Science, Ain Shams University, Cairo, Egypt; 2 Molecular Cancer Biology Group, Zoology Department, Faculty of Science, Ain Shams University, Cairo, Egypt; 3 Center of Excellence in Recombinant Pharmaceutical Proteins, Biochemistry and Molecular Biology Department, Theodore Bilharze Research Institute, Cairo, Egypt

**Keywords:** Plasmid, Miniprep, RNase A, DNA ladder

## Abstract

As a consequence of Covid-19 pandemic, the basic lab consumables are in shortage, especially in the low-income countries. Thus, the main objective of the present study is to develop and evaluate homemade solution to isolate plasmid. To pursue this objective, RNase A was overexpressed in Bl21 DE3 cells (*E. coli* strain) and prepared as crude refolding reaction with proper activity. Also, lysis buffers, neutralization buffer, and washing buffers were prepared. The homemade miniprep kit showed successful isolation of the px48SpCas9 plasmid. The prepared plasmid purity was enough to be used successfully in PCR amplification. In addition, to get extra benefits from this study, seven primers were designed to match the plasmid backbone to produce DNA ladder (100–1500 bp). In conclusion, we were able to have attainable working solutions for plasmid miniprep and DNA ladder.

## Introduction

Plasmids are DNA extrachromosomal genetic elements, which are found in most species of Eubacteria, Archae and Eukaryote. Plasmids replicate independently (Garcillan-Barcia et al. 2011). Molecular cloning is an essential experimental step in biology which serves different research fields including industry. Thus, isolation of plasmid DNA from microorganism is a crucial step in many molecular biology methods, which then can be used for characterization, cloning. Plasmid mini-Prep technique is used to isolate small plasmid DNA from micro-organism (Birnboim 1983; Clewell and Helinski 1969; Garcillan-Barcia et al. 2011).

Plasmid mini-Prep could be executed using many protocols. However, silica-based spin column is among the standard methods to isolate plasmid (Boom et al. 1990; Marko et al. 1982; Pronobis et al. 2016). The miniprep method is based on the alkaline lysis of the bacteria, removal of RNA using ribonuclease, selective precipitation of other molecules and plasmid adsorption on silica columns (Zhang and Cahalan 2007). In this type of miniprep kits, guanidine hydrochloride allows DNA dehydration which leads to binding of DNA phosphate groups to silica (Vogelstein and Gillespie 1979). Sodium hydroxide disrupts hydrogen bonds, leading to unwinding of both genomic and plasmid DNA. Then, neutralization step is applied where the small plasmid DNA could rewind but the genomic DNA fails to rewind. Centrifugation after neutralization step precipitates white insoluble materials which include genomic DNA, proteins, and lipids. The supernatant is applied to spin columns, in which plasmid DNA binds to silica then washed and eluted in pure form (Zhang and Cahalan 2007).

In our basic science research community, people did experience a big shortage in molecular biology consumables after covid-19 pandemic. Our lab is currently encountering delayed orders for several months, for instance ordering oligos takes 40–100 days to be delivered. In addition, we did experience a big rise in the price of basic molecular biology kits (2–3 folds). These issues could stop our research experiments and plans. Therefore, our main objective from the present work is to involve undergraduate students in the development of affordable working solutions which could save time and money for researchers during the crisis, especially in developing countries. Undergraduate students are our human resources that should take a part of the contemporary situation. Thus, this topic was designed for four students to pursue their graduation projects to create solution using old resources and in hand facilities to make this essential and crucial lab needs.

## Materials and methods

### Recombinant RNase A expression and evaluation

pET22b RNase A was a gift from Ronald Raines (Addgene plasmid # 58903; http://n2t.net/addgene:58903; RRID:Addgene_58903) (delCardayre et al. 1995). The RNase A expression plasmid was obtained from Addgene with T7 promoter, ampicillin resistance, and bovine pancreatic RNAase A gene ORF. The purchased plasmid was in DH5 alpha cells which are used for cloning and plasmid storage with minimum mutagenesis activity. GeneDireX miniprep kit (Simply™) was used to purify the plasmid from DH5 alpha cells. Around 50 ng of the purified plasmid was used to transform chemically prepared competent BL21 star DE3 (Invitrogen) *E.coli* strain, then plated on LB agar plates (containing 50 µg/ml Ampicillin). Selected colony was inoculated into a 10 ml LB medium containing ampicillin (50 μg/ml) and cultivated overnight at 37 °C. In the next day, the overnight culture was added to 200 ml selective cultures which have been induced by adding IPTG ( 5 mM) at OD600 0.6. After induction for 24 and 48 h, cultures were centrifuged at 7000 g and 4 °C and pellets were resuspended in 0.5 mL of PBS buffer and sonicated. The RNase expression was visualized using SDS-PAGE in comparison to uninduced control (El-Dabaa et al. 2022).

RNase A recombinant protein was mainly in inclusion bodies (IB). Therefore, inclusion bodies were purified using mechanical homogenizer and different kind of buffers (TNMFX-2 M Urea (50 mM tris-base, 150 mM NaCl, 1 mM EDTA, 2 M Urea, PH 8), PBST, 8 M urea, and TNMFX-0.1% Triton X100) following a protocol from Proteintech, Japan (www.ptglab.com). Purified inclusion bodies were solubilized in 8 M urea solution containing 0.2 M Tris–HCl, pH 8.5 and 0.1 M (β-mercaptoethanol) for protein reduction. The denatured protein was refolded using rapid dilution in refolding buffer (30 mM Tris–HCl, pH 8.5, 0.5 mM oxidized glutathione, 3 mM reduced glutathione, 0.4 M L Arginine) (Futami et al. 2000). To test the activity of RNase A, crude RNase refolding reaction and RNase inclusion bodies (as control with non-folded, non-reactive RNase) were incubated with total RNA purified from HepG2 cells. The crude refolded RNase was used directly in the plasmid miniprep as described later.

### Plasmid miniprep solutions preparation

The main composition of buffers was retrieved from online OpenWetware (https://openwetware.org/wiki/Qiagen_Buffers) and composed with slight modifications. The solutions were prepared as the following: P_1_ lysis Buffer (50 mM Tris–HCL PH 8, 10 mM EDTA), P2 buffer (200 mM NaOH, 1% SDS), N3 neutralization buffer (4.2 M GuHCL, 0.9 M CH_3_COOK, pH 4.8), PB buffer (5 M Gu-HCL, 30% ethanol), and PE buffer (10 mM Tris–HCL pH 7.5, 80% EtOH). EZ-10 spin columns were purchased from BioBasic, Canada.

### Miniprep protocol

#### Lysis

1. 1.2 ml of transformed bacterial culture was harvested by centrifugation at 4000×*g*.
2. The bacterial pellet was resuspended in 250 µl of P_1_ lysis buffer and 5 µl of crude refolded RNase.
3. 250 µl of P_2_ buffer was added and mixed by inverting for 5–7 times to get a clear solution (do not incubate it more than 5 min).
4. 350 µl of N_3_ buffer was added and inverted immediately for 5–7 times, followed by centrifugation for 10 min at 13000×*g*.
5. 750 µl from supernatant of the previous step was applied to a spin column tube then centrifuge for 30–60 secs at 10000×*g*. Then the flow-through was discarded.

#### Washing

1. The spin column was washed by 500 µl of PB buffer then centrifuged for 30–60 secs at 10,000×*g*, followed by discarding the flow-through.
2. 750 µl of PE buffer was added to the spin column and centrifuged for 30–60 secs at 10000×*g*.
3. After discarding the flow-through, to get rid of any washing contaminants, an additional centrifugation step at 10000×*g* for 1 min at full speed was executed.

#### Elution

1. The spin column was transferred into clean 1.5 ml microcentrifuge tube.
2. 30-50 µl of ddH_2_O was added and incubated for 5 min at room temperature, then was centrifuged for 1 min at full speed.
3. The previous step was repeated for one more time.
4. The eluted plasmid was stored at −20 °C.

### Validation

#### Plasmid miniprep preparation

To test our homemade solutions, DH5 alpha cells were transformed with of px48SpCas9 (1.1). The plasmid which was kindly provided by Ole M. Seternes (UiT, Norway). Then, our miniprep solutions were utilized in comparison with commercial kit from GeneDirex. The extracted plasmids were evaluated by electrophoresis on 1.5% agarose gel and visualized by UV-transilluminator.

#### PCR amplification

In addition, for extra validation of prepared plasmid purity, a PCR was done using specific primers for this plasmid. (LD F: 5′ acggatcgacctgtctcagc 3′ and LD300 R: 5’ ccggtggtgcagatgaactt 3′).

### Homemade DNA Ladder preparation

The purified px48SpCas9 (1.1) plasmid was used as a PCR template to make DNA ladder. Primers were designed to match the plasmid sequence to give 7 PCR products ranging from 100 bp to 1.5 kb (Table1). Gradient temperature ranging from 57 to 67 °$\mathrm{C}$ was tested to optimize each PCR fragment. The gradient range of annealing temperature was selected ± 5 °C of melting temperature of the synthesized primers.

**Table 1:** Primers used for PCR amplification of DNA ladder different fragments

Primer name	Sequence
LD F	5′ acggatcgacctgtctcagc 3′
LD100 R	5′ gccctctccactgccgaatt 3′
LD200 R	5′ gctcgaccaggatgggcacc 3′
LD300 R	5′ ccggtggtgcagatgaactt 3′
LD400 R	5′ gaagaagtcgtgctgcttca 3′
LD500 R	5′ tgtcgccctcgaacttcacc 3′
LD800 R	5′ tgtgatcgcgcttctcgttg 3′
LD1500R	5′ ggaaccctaaagggagcccc 3′

## Result

### RNase A was expressed in BL21 (*E. coli* stain) and prepared in active form

**Fig. 1:**
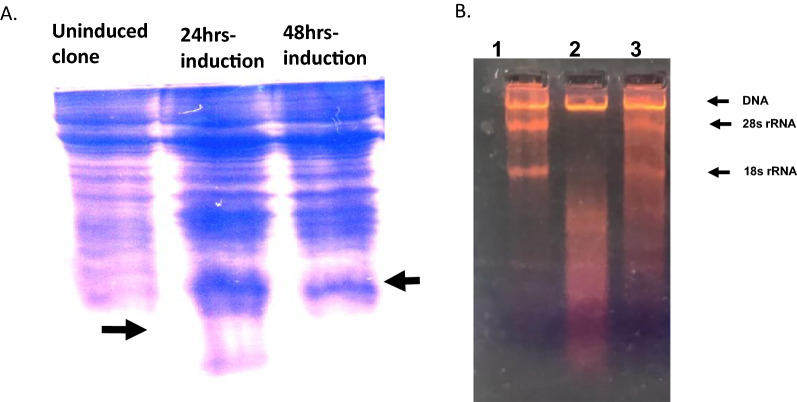
Expression and evaluation of prepared recombinant RNase A. **A** 15% SDS-PAGE of cell lysates for detection of recombinant RNase A protein expression in BL21 (DE3) cells after IPTG induction. Lane (1) non-induced bacteria lyasate. Lanes (2 and 3): Bacteria lysates after induction for 24 and 48 h. Arrows indicate RNase band. **B** Evaluation of the activity of the prepared crude recombinant RNase A using Total RNA from HepG2 cells. Lane (1): Total RNA without the RNase A. Lane (2) Total RNA after incubation with the prepared recombinant RNase A. Lane 3 after addition of the RNase inclusion bodies

RNase A overexpression was detected using 15% SDS-PAGE in comparison with un-induced cultures as shown in Figure 1A. Additionally, the activity of RNase A in refolding solutionshowed proper total RNA degradation activity in comparison with non-folded inactive RNase in inclusion bodies (Figure 1B).

### Homemade miniprep kit did successfully purify plasmid from transformed bacteria

**Fig. 2:**
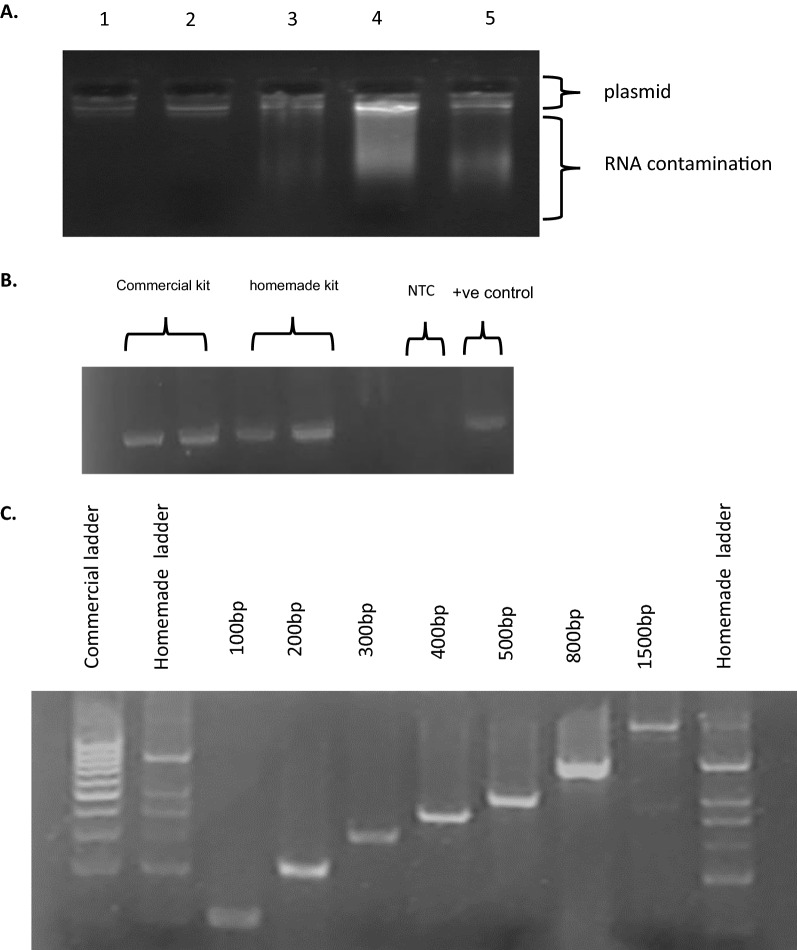
Validation of the home made miniprep kit. **A** Plasmid miniprep using the home-made kit in comparison with commercial kit. Lanes (1 and 2): plasmid minipreps using the GeneDirex commercial kit. Lane (3) plasmid miniprep using our home-made kit with RNase. Lane (4): plasmid miniprep using our home-made kit with RNase inclusion bodies. Lane (5) homemade kit with half amount of RNase A. **B** Agarose gel electrophoresis of px48SpCas9 plasmid minipreps PCR products: Lane (1 and 2): PCR for plasmid minipreps made using the commercial kit. Lanes (3 and 4): PCR for plasmid minipreps made using the home-made kit. Lanes (5 and 6) Negative and positive controls. **C** Agarose gel electrophoresis for DNA ladder PCR amplified fragments. Lane (1): commercial DNA ladder. Lanes (2 and 10): The home-made DNA ladder (mixture of the PCR amplified fragments. Lanes (3 to 9): PCR amplification for each fragment 100 bp, 200 bp, 300 bp, 400 bp, 500 bp, 800 bp and 1500 bp

The prepared plasmid miniprep kit was prepared according to the available data online and compared to the commercial GeneDirex kit. In our homemade kit, RNase crude refolding reaction was included in P1 lysis buffer as non-purified enzyme. To verify the extraction procedure, the purified plasmid preps were subjected to electrophoresis on 1.5% agarose gel as shown in Fig. 2A. The figure shown successful purification of the plasmid with our homemade kit with small traces of remaining RNA.

As further confirmation of the purity of the plasmid minipreps made by the developed kit and its suitability for PCR amplification, the PCR using specific primers for the plasmid preps showed successful amplification from minipreps prepared using our homemade kit and the commercial one (Fig. 2B).

### Amplification of different fragments from the purified plasmid to make homemade DNA ladder

As a downstream application, homemade DNA ladder was made by PCR amplification using seven primers matching px48SpCas9 (1.1) and the purified plasmid as a template. The purified amplicons were mixed to make DNA ladder and electrophoresed as shown in Fig. 2C.

## Discussion

At the end of 1990s and beginning of this century, most of the researchers were making their own solutions by mixing raw materials in the lab. Meanwhile, the biotechnology companies started to offer ready-made solutions/kits to enhance and facilitate working procedure in the research laboratories. At the beginning, molecular biology products were very expensive. However, after onset of many biotechnology enterprises, the reagents were distributed worldwide in affordable prices. The last two years after covid-19 pandemic, the situation has been globally changed, particularly in the developing countries. These kits became very expensive and even if we have enough budget, placing an order takes many months to get the required kits, which highly entangle our research schedules. The previous reasons were behind our motivation to find home-made solution.

Spin-based nucleic acid purification is the most common used format for DNA/RNA/plasmid isolation (Abdelfattah et al. 2022; Birnboim 1983; Clewell and Helinski 1969; Garcillan-Barcia et al. 2011). For plasmid miniprep, composing the whole kit setting in affordable format needs silica columns, purification solutions, and recombinant RNase A. Hereby, the solutions composition was retrieved, The overexpression of recombinant RNase A was performed and the crude refolded RNase A activity was tested against total RNA purified from HepG2 cells. Accordingly, we did include the crude refolded RNase in the lysis buffer of the homemade kit.

Validation of the developed miniprep kit for plasmid purification in comparison to a commercially available kit proved sufficient performance concerning the yield and purity. However, some RNA contamination was still visualized. This could be due to the high dilution of the RNase in the refolding buffer or interfering from other contaminants in the crude enzyme preparation. But fortunately, this did not interfere with DNA PCR amplification from these minipreps, which revealed enough purity for this purpose and exclude the need for further expensive and tedious chromatographic purification of the RNase A enzyme.

DNA ladder is one of the essential standard reagents in molecular biology laboratories (Cooney 1994; Parker et al. 1977). DNA ladder could be made by restriction enzyme digestion, PCR amplification, or combination of both (Henrici et al. 2017; Lan et al. 2012; Murray and Monchawin 1994; Wang et al. 2011). To utilize the isolated plasmid, seven pair of primers were designed to amplify different sizes of amplicon. After mixing the different PCR products, a simple DNA ladder was obtained with seven bands ranging from 100 to 1500 bp bands.

In conclusion, homemade plasmid miniprep kit have been prepared and validated with sufficient performance using affordable and established resources with simple steps which led to time and budget savings. In addition, plasmid was used to make a simple DNA ladder.

## Limitations

In this work, the home-made plasmid miniprep kit showed a small contamination of RNA with plasmid minipreps. This may be due to several factors like the high dilution of the crude RNase preparation or the low purity of the enzyme and may also the non-optimized refolding of the RNase. But we did not optimize or check these factors because the performance of this kit is sufficient in comparable to the commercial one concerning yield, purity and suitability for PCR which is satisfactory for many purposes like transformants screening for gene cloning, protein expression, detection of plasmid related traits, etc. Also, chromatographic purification, using more enzymes and further optimization of folding will require more budget and more time and will add complexity to the home-made kit preparation.

## Data Availability

The datasets used and/or analysed during the current study are available from the corresponding author on reasonable request.

## Abbreviations

DNA: Deoxyribonucleic acid
RNA: Ribonucleic acid
PCR: Polymerase chain reaction
